# Telomerase inhibition by siRNA causes senescence and apoptosis in Barrett's adenocarcinoma cells: mechanism and therapeutic potential

**DOI:** 10.1186/1476-4598-4-24

**Published:** 2005-07-15

**Authors:** Masood A Shammas, Hemanta Koley, Ramesh B Batchu, Robert C Bertheau, Alexei Protopopov, Nikhil C Munshi, Raj K Goyal

**Affiliations:** 1 Center for Swallowing and Motility Disorders, VA Boston Healthcare System, Boston, MA 02132, USA; 2 Research and Development, VA Boston Healthcare System, Boston, MA 02132, USA; 3 Adult Oncology, Dana Farber Cancer Institute, Boston, MA 02115, USA; 4 Medicine, Beth Israel Hospital, Boston, MA 02115, USA; 5 Medicine, Harvard Medical School, Boston, MA 02115, USA

**Keywords:** SiRNA, Telomerase, Telomere, Barrett's esophageal adenocarcinoma, Senescence, Apoptosis, Cancer treatment, Cancer prevention, P73, P63

## Abstract

**Background:**

In cancer cells, telomerase induction helps maintain telomere length and thereby bypasses senescence and provides enhanced replicative potential. Chemical inhibitors of telomerase have been shown to reactivate telomere shortening and cause replicative senescence and apoptotic cell death of tumor cells while having little or no effect on normal diploid cells.

**Results:**

We designed siRNAs against two different regions of telomerase gene and evaluated their effect on telomere length, proliferative potential, and gene expression in Barrett's adenocarcinoma SEG-1 cells. The mixture of siRNAs in nanomolar concentrations caused a loss of telomerase activity that appeared as early as day 1 and was essentially complete at day 3. Inhibition of telomerase activity was associated with marked reduction in median telomere length and complete loss of detectable telomeres in more than 50% of the treated cells. Telomere loss caused senescence in 40% and apoptosis in 86% of the treated cells. These responses appeared to be associated with activation of DNA sensor HR23B and subsequent activation of p53 homolog p73 and p63 and E2F1. Changes in these gene regulators were probably the source of observed up-regulation of cell cycle inhibitors, p16 and GADD45. Elevated transcript levels of FasL, Fas and caspase 8 that activate death receptors and CARD 9 that interacts with Bcl10 and NFKB to enhance mitochondrial translocation and activation of caspase 9 were also observed.

**Conclusion:**

These studies show that telomerase siRNAs can cause effective suppression of telomerase and telomere shortening leading to both cell cycle arrest and apoptosis via mechanisms that include up-regulation of several genes involved in cell cycle arrest and apoptosis. Telomerase siRNAs may therefore be strong candidates for highly selective therapy for chemoprevention and treatment of Barrett's adenocarcinoma.

## Background

Senescence and apoptosis normally counteract cancer development and ability of cancer cells to disrupt these processes is 'lifeline' of cancer [1]. Oncogenes such as ras and myc can not induce oncogenesis unless intracellular mediators of senescence and apoptosis are disrupted. Most anticancer agents act by stimulating intracellular mechanisms for cellular senescence and apoptosis; they do not simply destroy them directly. The ability of these drugs to reactivate the normal or activate alternate intracellular signals for replicative senescence and apoptosis in cancer cell determines the sensitivity and efficacy of the anticancer drugs [1].

One of the mechanisms by which cancer cells bypass normal cellular senescence is the elevated expression of the enzyme telomerase that replicates telomeric DNA [2]. Telomeres are tandem repeats of six-nucleotide sequence (TTAGGG) that protect the ends of chromosomes from being recognized as damaged DNA. Normally, during cell division telomeres shorten because DNA polymerase that replicates all DNA, is unable to copy telomeric DNA distal to the site of last primase. In normal somatic cells telomeres progressively shorten as 50–100 base pair telomeric DNA is lost with each round of cell division. When the telomeres reach critical shortening, DNA damage is sensed by DNA sensing molecules that activate intracellular processes that lead to irreversible cell cycle arrest and replicative senescence [3]. The replicative senescence limits the potential of somatic cells for population doubling and hence limits their growth [4].

Telomere length can be preserved by an enzyme, telomerase. Telomerase contains a catalytic unit with reverse transcriptase activity (hTERT) and an RNA part that provides template for telomere extension [2]. Telomerase is normally expressed only in stem cells such as those found in hematopoeitic tissues, gastrointestinal and skin epithelium and germ-line cells but is nearly absent in most somatic cells [5]. However, approximately 90% of cancers express high levels of telomerase activity [5]. Induction of telomerase activity bypasses normal cellular senesce in cancer cells and endows them with unlimited replicative potential which is one of the key features of all cancer cells. Suppression of telomerase activity in cancer cells may reactivate telomere shortening. However, such telomere shortening may be more acute and may lead to acutely inducible form of cellular senescence [1]. Suppression of telomerase activity has also been reported to cause apoptosis of cancer cells. Although, normally, senescent cells may be resistant to apoptosis, chemical inhibitors of telomerase have been shown to cause replicative senescence as well as apoptosis in cancer cells [6-8].

Signaling pathways involved in reactivation of senescence and apoptosis associated with telomerase inhibition in cancer cells are not fully understood. The signaling molecule, p53, that mediates cell cycle arrest and apoptosis in normal ageing is also considered important in inducing cell cycle arrest following telomere shortening. However it may be genetically or otherwise deleted or rendered ineffective in cancer cells. Recently two P53-related genes, P73 and P63 (also known as p73alphaL, p63alpha, p40, TP51, KET and AIS) with striking sequence homology to P53 have been identified [9]. The actions and regulation of p73 and p63 and their isoforms are complex and their targets may be different than those of p53. In the past it was generally believed that cellular stress and DNA damage induce only p53. However it has now been shown that p73α is the mediator of p53-independent, DNA damage induced, cell cycle arrest and apotosis [9,10]. DNA damage associated with telomerase shortening may also activate other signaling pathways that lead to cell cycle arrest and apoptosis.

Several approaches have been proposed to achieve telomerase suppression. These approaches include: anti-sense oligonucleotides, targeting RNA component of telomerase [7], chemical inhibitors of telomerase [11], small molecule pharmaceuticals that target hTERT [12], and telomerase vaccine [13]. Chemical inhibitors of telomerase may also exert side effect whereas molecular antagonism such as with siRNAs should produce selective suppression of telomerase, however, the effectiveness or feasibility of this approach has not been reported.

Esophageal adenocarcinoma arises from specialized intestinal metaplasia of the Barrett's esophagus which is a complication of gastro-esophageal reflux disease [14]. Esophageal adeno-carcinoma has a rapidly rising incidence, poor response to chemotherapy and overall poor survival rate [15]. In development of this cancer, intestinal metaplasia gradually progresses through different stages of dysplasia to invasive adenocarcinoma [14]. Telomerase activity is elevated in the metaplastic cells [16] and is further elevated in the adenocarcinoma [7,16]. Therefore, telomerase inhibition may have potential in treatment and chemprevention of esophageal adenocarcinoma.

The purpose of the present studies in Barrett's associated adenocarcinoma SEG-1 cells was: 1) to examine the feasibility and effectiveness of telomerase siRNAs to inhibit telomerase; 2) to examine whether selective telomerase inhibition leads to senescence and apoptosis; and 3) to identify signaling pathways of telomerase inhibition induced senescence and apoptosis in cancer cells.

We found that telomerase siRNAs targeted against two regions of the human telomerase, caused efficient inhibition of telomerase expression, loss of telomerase activity and severe telomere shortening. Telomere shortening was associated with senescence and apoptotic cell death. Gene expression studies revealed that telomerase inhibition was associated with elevated expression of p73, p63, and E2F1. Elevated transcript levels of genes mediating cell cycle arrest such as p21, p16 and GADD45 and those implicated in apoptosis such as FasL, Fas, caspase 8, proapototic CARD 9, caspases 7 and 3 were also observed.

## Results

### SiRNA uptake and loss of telomerase expression

Cy-3 labeled control RNA duplex (Dharmacon) at 50 nM was introduced into cells using different transfection reagents and methods. Delivery of siRNA by *Trans*IT-TKO reagent (Mirus) resulted in the best transfection efficiency 81 ± 3% (data not shown). Amount of siRNA delivered per cell was also considerably higher following transfection with this reagent.

**Figure 1:**
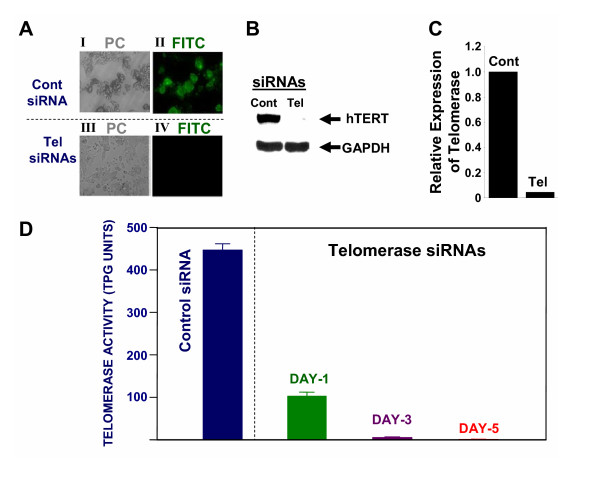
**Effect of telomerase-specific siRNAs on telomerase expression and activity in SEG-1 cells. **(**A**). Control (Cont) or telomerase specific (Tel) SiRNAs were transfected into Barrett's associated adenocarcinoma cells (SEG-1) and the cells were analyzed for protein levels of telomerase at day 7 by immunostaining, using a rabbit polyclonal antibody against telomerase. Antigen-antibody complex was located by incubation of cells with FITC-labeled anti-rabbit secondary antibody. Transfected cells within the same microscopic field were viewed and photographed by phase contrast (PC) or by fluorescence emitted at 518 nm (FITC filter). Using the FITC filter, telomerase positive cells appear bright green. (**B**). SEG-1 cells were treated exactly as described for panel "A" and telomerase expression was monitored by western blot analysis using a rabbit polyclonal anti-telomerase antibody. (**C**). Bar graph showing relative expression of telomerase in control (Cont) or telomerase (Tel) siRNA treated SEG-1 cells, as assessed by western blot analysis (shown in panel B). (**D**). SEG-1 cells were transfected with control (Cont) or telomerase specific (Tel) SiRNAs and evaluated for telomerase activity at days 1, 3, and 5 after transfection. Telomerase activity was evaluated using TRAPEZE^R ^XL Telomerase Detection Kit, a highly sensitive, quantitative, and non-isotopic version of the original Telomeric Repeat Amplification Protocol. Briefly the cell lysates (1000 cell-equivalents) were mixed with TRAPEZE^R ^XL reaction mix containing Amplifuor™ primers, incubated for 30 min at 30°C, and telomerase products generated by PCR were quantitated using a Fluorescence Plate Reader. Telomerase activity in SEG-1 cells treated with control siRNAs for 5 days and the cells treated with telomerase specific siRNAs for days 1, 3, and 5, is shown in TPG (total product generated) units.

Utilizing *Trans*IT TKO transfection reagent (Mirus, Madison, WI), telomerase specific and control (siControl; Dharmacon) siRNAs were introduced into SEG-1 cells. On day 7, the transfected cells were evaluated for telomerase protein by immunofluorescence and western blot analyses. For immunofluorescence, the cytospins of transfected cells were fixed and incubated with rabbit polyclonal antibody to telomerase. Antigen-antibody complex was detected by incubation with a fluorescein isothiocyanate (FITC)-labeled secondary antibody. Telomerase expression, seen as green fluorescence in SEG-1 cells treated with control siRNA (Figure 1A, panel II), is not detected in cells treated with telomerase specific Tel-siRNAs (Figure 1A; panel IV). SiRNA mediated inhibition of telomerase expression was also confirmed by western blot analysis using a polyclonal anti-telomerase antibody. Consistent with immunofluorescence, the expression of telomerase in cells transfected with Tel-siRNAs was reduced 95% relative to the cells treated with control siRNAs (Figure 1B and 1C).

### SiRNA mediated inhibition of telomerase activity

BEAC cells (SEG-1) were treated with telomerase specific (Tel) siRNAs for different durations, using *Trans*IT-TKO reagent. Telomerase activity was assessed using TRAPEZE^R ^XL Telomerase Detection Kit which provides a highly sensitive, quantitative, and non-isotopic version of the original TRAP (Telomeric Repeat Amplification Protocol) assay. The kit utilizes "Fluorescence Energy Transfer" primers to generate fluorescently labeled TRAP products which can be quantitated by a fluorescence plate reader. The modifications of primer sequence and internal PCR control added in this assay allow a more accurate quantitation of telomerase activity *in vitro*. Telomerase activity was assessed in lysates (1000 cell-equivalents) of SEG-1 cells treated with control or Tel-siRNAs. Telomerase activity in cells transfected with Tel-siRNAs was reduced 77 ± 3%, as early as day 1 (Figure 1D). A complete inhibition of telomerase activity was observed in these cells at day 3. Control siRNA had no effect on telomerase activity in SEG-1 cells (Figure 1D).

### Inhibition of SEG-1 cell growth

**Figure 2:**
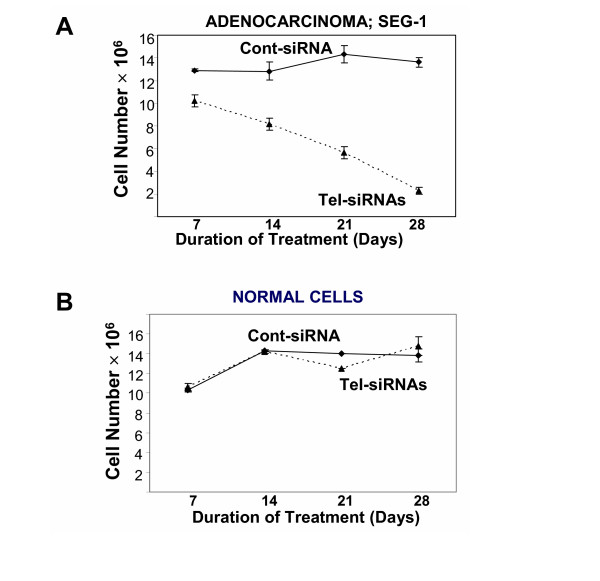
**Limited replicative potential of BEAC cells treated with telomerase specific SiRNAs. **Barrett's associated adenocarcinoma cells SEG-1 (**A**) or normal cells from stomach and intestine (CRL7869) (**B**) were transfected with control (◆) or telomerase specific (▲) siRNAs and cultured for four weeks. At the end of each 7-day treatment cycle, cells were harvested and the number of viable cells was determined using trypan blue exclusion.

SEG-1 cells were transfected weekly with control or telomerase-specific siRNAs and substrate-attached viable cell number was counted. A marked inhibition of cell proliferation was observed in SEG-1 cells in all three treatments with Tel-siRNAs. Viable cell number did not change for the first seven days, and then gradually declined by 77–85% by 28 days (Figure 2A). Exposure of normal (CRL7820) cells to siRNA for four weeks had no effect on cell viability (Figure 2B).

### Telomere shortening by telomerase siRNAs

**Figure 3:**
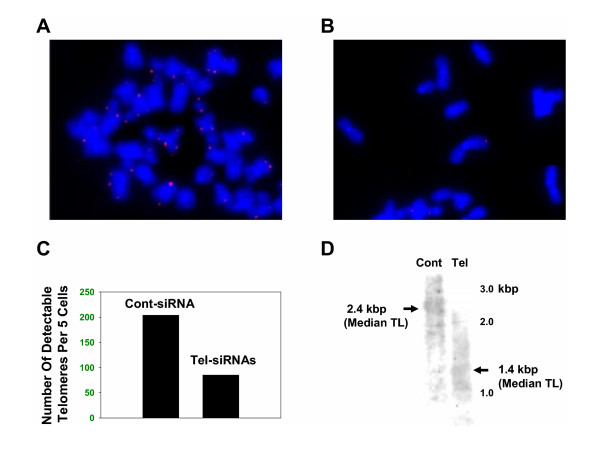
**Reduction in telomere length following siRNA treatment of BEAC cells. **SEG-1 cells were transfected with control or telomerase specific siRNAs and telomere length was analyzed either by fluorescent in situ hybridization of metaphase chromosomes, using the Cy3-PNA (C_3_TA_2_)_3 _probe (A, B, C) or by southern hybridization of genomic DNA to a telomere-specific probe (D). (**A**) SEG-1 cells were treated with control siRNAs for 3 weeks and telomeres on individual chromosomes were analyzed using Telomere-FISH, as described. Telomeres shown as red dots can be seen on most, if not all chromosomes. (**B**) SEG-1 cells were treated with telomerase specific (Tel) siRNAs for 3 weeks and telomeres were analyzed using Telomere-FISH. Majority of chromosomes are without any telomeres (absence of red dots). The chromosomes which have telomeres (red dots), have a relatively weaker signal, indicative of shorter telomeres; (**C**) Bar graph showing number of telomeres (red dots) found per 5 cells analyzed at random. (**D**) SEG-1 cells, transfected with control (Cont) or telomerase specific (Tel) siRNAs, were cultured for 3 weeks. Genomic DNA was isolated and median telomere length was determined by southern hybridization, as described. Median Telomere Length (TL) for each treatment is indicated by arrows.

We next analyzed telomere length following exposure of SEG-1 cells to telomerase specific siRNAs, for 3 weeks. The effect of telomerase inhibition on telomere length of individual chromosomes was evaluated by Telomere-FISH (Figure 3A, B, and 3C). Telomeres could be seen as red dots at the ends of almost all chromosomes, in cells treated with control siRNA (Figure 3A). However in cells treated with telomerase siRNAs, 60% chromosomes appeared to have telomere-free ends, indicating complete erosion of telomeric ends on these chromosomes (Figure 3B and 3C). "Telomere length", the mean size of telomeric restriction fragments generated by digesting the DNA with telomere-sparing restriction endonucleases was also estimated using genomic DNA isolated for each cell sample [17]. Consistent with Telomere-Fish data, the exposure of SEG-1 cells to telomerase specific siRNAs for 3 weeks, led to a marked (1000 bp) reduction in median telomere length (Figure 3D).

### siRNA mediated telomerase inhibition leads to senescence

**Figure 4:**
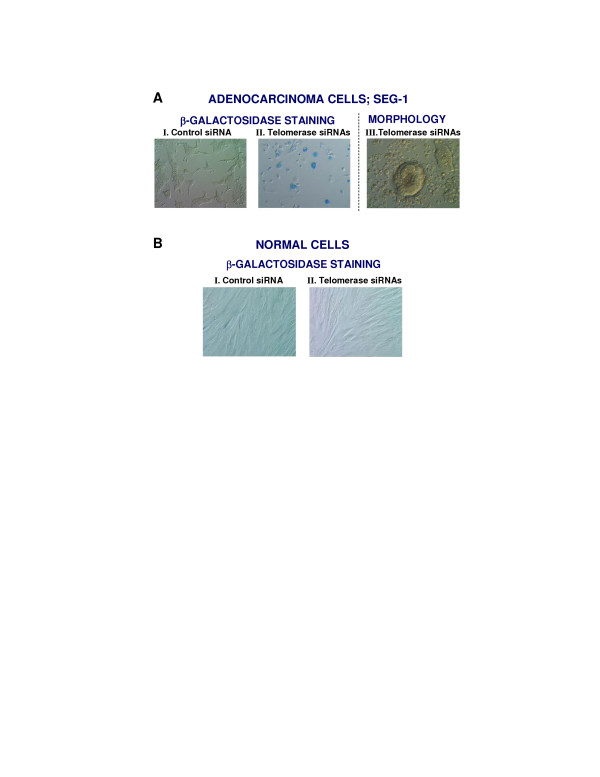
**Senescence following treatment of SEG-1 cells with telomerase specific siRNAs. **SEG-1 cells were transfected with either control or telomerase specific siRNAs and cultured for three weeks. For senescence assay, cells were rinsed in PBS and stained overnight in solution containing X-gal. Expression of β-galactosidase was monitored using an Olympus^® ^fluorescence microscope. Panels: **A I**. SEG-1 cells transfected with control siRNA, evaluated for of β-galactosidase expression. **A II**. SEG-1 cells transfected with telomerase siRNA, evaluated for of β-galactosidase expression. Expression of β-galactosidase is seen as blue colour. **A III**. Typical senescence associated morphology in a subset of SEG-1 cells transfected with telomerase siRNAs. **B I**. Normal cells from stomach and intestine, transfected with control siRNA and evaluated for of β-galactosidase expression. **B II**. Normal cells from stomach and intestine, transfected with telomerase specific siRNAs and evaluated for of β-galactosidase expression.

SEG-1 cells treated with siRNAs for three weeks were harvested and analyzed for senescence associated β-galactosidase expression, as described above. Senescence, as indicated by β-galactosidase staining (blue colour), was detected in 41 ± 6% cells transfected with Tel-siRNAs (Figure 4A, panel II). A subset of telomerase siRNA treated SEG-1 cells also showed typical senescent morphology (Figure 4A; panel III). Senescent cells were not seen in SEG-1 cells treated with control siRNAs (Figure 4A; panel I) or in non-cancer cells treated with control or telomerase specific (Tel) siRNAs (Figure 4B).

### Induction of apoptosis following siRNA treatments

**Figure 5:**
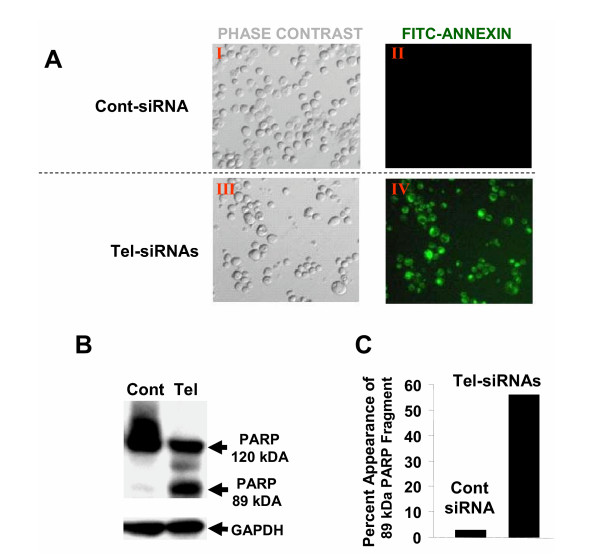
**Apoptosis following treatment of BEAC cells with telomerase siRNAs. **SEG-1 cells, transfected with control (Cont) or telomerase specific (Tel) siRNAs were cultured for three weeks and analyzed for apoptosis by evaluating annexin labeling or cleavage of PARP. **A. **Cells were mixed with annexin V-BIOTIN and treated sequentially with streptavidin conjugated to fluorescein isothiocyanate (FITC) and propidium iodide (PI). Apoptotic cells within the same microscopic field were viewed and photographed by phase contrast (**PC**) or by fluorescence emitted at 518 nm (**FITC **filter). Using the FITC filter, early apoptotic cells (positive for Annexin V-Biotin-FITC staining) appear bright green. **B. **SEG-1 cells were treated exactly as described for panel A and analysed for cleavage of poly(ADP-ribose) polymerase (PARP), a marker for apoptosis. PARP was identified by western blotting using a rabbit polyclonal antibody against PARP (Santa Cruz). **C. **Bar graph showing percentage of PARP found to be cleaved in cells treated with control or telomerase siRNAs.

SEG-1 cells treated with control or telomerase specific siRNAs for three weeks were evaluated for annexin V labeling, a marker for early apoptosis. Cells treated with Tel-siRNAs led to > 80% cells in apoptosis, as detected by annexin V staining (Figure 5A, panel V). Less than 2% cells were labeled with annexin in untreated SEG-1 samples.

The siRNA treated cells were also evaluated for poly(ADP-ribose) polymerase (PARP), a protein which undergoes caspase-3 mediated cleavage into 89- and 24-kDa fragments during apoptosis. As shown in Figure 5 (panels B and C), the PARP was mostly intact in control siRNA treated cells with only 2% cleaved, as indicated by appearance of 89 kDa fragment (Figures 5B and 5C). However in the cells treated with telomerase specific (Tel) siRNAs, a major fraction (55%) of PARP appeared as cleaved (89 kDa) fragment thus confirming the induction of apoptosis following telomerase inhibition.

### Effect of telomerase inhibition on gene expression profile

**Figure 6:**
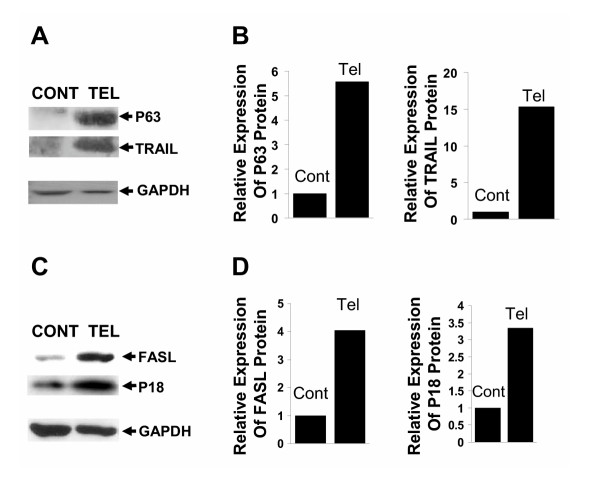
**Induction of apoptosis proteins following treatment of SEG-1 cells to telomerase specific siRNAs. **SEG-1 cells, transfected with control (Cont) or telomerase specific (Tel) siRNAs were cultured for two weeks and analyzed for protein expression by western blotting. The expression of p63, TNF-related apoptosis inducing ligand (TRAIL), TNF ligand superfamily, member 6 (FASL), and p16 was monitored using mouse monoclonal anti-p63 (Zymed), rabbit polyclonal anti-TRAIL (Santa Cruz), mouse monoclonal anti-FASL (Cell Science), and mouse monoclonal anti-p16 (Sigma) antibodies, respectively. **A. **Western blot showing expression of p63 and TRAIL in control (Cont) and telomerase (Tel) siRNA treated cells. **B. **Bar graph showing relative expression of p63 and TRAIL following normalization with glyceraldehyde-3-phosphate dehydrogenase (GAPDH). **C. **Western blot showing expression of FASL and p16 in control (Cont) and telomerase (Tel) siRNA treated cells. **D. **Bar graph showing relative expression of FASL and p16 following normalization with glyceraldehyde-3-phosphate dehydrogenase (GAPDH).

SEG-1 cells were transfected with telomerase specific (Tel) siRNAs. After 2 weeks, the cells were harvested, total RNA isolated, and gene expression profile was analyzed using U133 arrays (Affymetrix) representing 33,000 human genes, as described previously [7]. Gene expression values of independently conducted experiments showed excellent correlation (R^2 ^0.98; not shown). SiRNA treatment resulted in a ≥2-fold change in 600 out of 33,000 genes surveyed. Changes in some of the known genes involved in DNA damage sensing, cell cycle arrest and apoptosis are shown (see Additional file 1). Telomere shortening following siRNA mediated telomerase suppression produced a ≥2-fold up-regulation of genes for DNA damage recognition and DNA repair protein HR23B; p53 family proteins p73 and p63 and their regulators, E2F1 and MDM2; cell cycle inhibitors p21, p16 and GADD 45; and pro-apoptotic proteins, FasL, Fas, and CARD9. There was also a 1.5 fold increase in the gene expression of caspase 8, caspase 7, and caspase 3. A subset of expression changes were also confirmed by western blot analysis (Figure 6). Consistent with gene expression data, protein products of p63, FASL, and p16 were also elevated = 2-fold in SEG-1 cells treated with telomerase siRNAs relative to those treated with control siRNAs. Although not detected by gene expression profiling, western blot analysis also indicated a marked upregulation of tumor necrosis factor (ligand) superfamily, member 10 (TRAIL) (Figure 6), a protein which specifically induces apoptosis in transformed and tumor cells, but not in normal cells.

## Discussion

This study shows that in Barrett's adenocarcinoma SEG 1 cells, siRNAs targeting human telomerase can be efficiently delivered and results in a rapid inhibition of telomerase activity. The inhibition of telomerase activity is associated with: 1) a complete erosion of telomeres of majority of chromosomes and growth arrest of the cancer cells without affecting the normal cells from stomach and intestine; 2) induction of both senescence and apoptosis; and 3) up-regulation of key regulatory genes involved in cell cycle arrest and apoptosis.

RNA interference has emerged as a method for effective and specific inhibition of gene expression both *in vitro *and *in vivo *[18,25]. This study shows that a mixture of siRNAs directed against two different regions of telomerase produced near complete silencing of telomerase function along with suppression of the protein. The mixture of siRNAs in nanomolar concentration led to 77 ± 3% inhibition of telomerase activity within 24 hours and near complete inhibition in 72 hours.

Inhibition of telomerase activity was associated with a marked reduction (30% of the control) in median telomere length. The effect of RNAi-mediated inhibition of telomerase activity on telomere length has not been reported before. However, other inhibitors of telomerase have been shown to produce similar median reduction in telomere length. The estimation of median telomere length, however, does not provide information on telomere length on individual chromosomes that possess telomere of different lengths. Loss of all telomeres in a small number of chromosomes may trigger a DNA damage response when median telomere length is not critically reduced. In order to determine loss of telomeres on individual chromosomes, we hybridized metaphase chromosome spreads of treated and untreated cells with Cy3-PNA (C_3_TA_2_)_3 _probe. We found that in siRNA treated cells, 60% of chromosomes had no telomere signal, indicating complete erosion of telomeres on these chromosomes. These data suggest that the siRNAs produced potent inhibition of telomerase activity and loss of telomeres on majority of the chromosomes.

SiRNA mediated telomerase inhibition also resulted in cell death of >80% cancer cells in four weeks. The long delay of four weeks that was required for induction of cell death is consistent with the fact that loss of telomerase caused a relatively gradual telomere shortening. A telomere length below critical ~2 kbp [19], has been reported to result in growth arrest [20]. The growth arrest was associated with replicative senescence and apoptosis.

Telomere shortening induces senescence [21] which is permanent and irreversible cell cycle arrest in G1 phase thereby halting growth. Senescent cells express β-galactosidase and develop a characteristic flat and vacuole-rich cytoplasmic morphology, but remain metabolically active and are eliminated by cell necrosis and phagocytosis [22]. However, more recently senescent cells have been shown also to undergo p73 mediated apoptotic cell death in cancer cells. Telomerase inhibitors have been reported to cause replicative senescence in cancer cells [6]. Telomerase gene suppression by siRNAs induced β-galactosidase expression in 41 ± 6% and typical senescent phenotype in a subset (10 ± 4%) of the cells.

Telomerase inhibition with the siRNA mixture also induced apoptosis in 86 ± 5% of the cancer cells. Apoptosis was confirmed by both the annexin V labeling and PARP cleavage. Since molecular suppression of telomerase by gene silencing using siRNA is highly selective, these studies show that apoptosis is specifically due to telomerase inhibition. Although, chemical inhibitors of telomerase have also been reported to cause apoptosis [7], it was not clear whether they produced apoptosis specifically due to telomerase suppression or due to their unrelated side effects. However, induction of both senescence and apoptosis has also been observed following specific inhibition of telomerase by oligo-nucleotides targeting RNA component of telomerase.^6 ^It has been suggested that telomerase inhibitors may cause apoptosis due to severe and precipitous telomere shortening that may be associated with chromosomal rearrangement and DNA fusion. This condition termed 'crisis' may be associated with activation of apoptotic pathways resulting in apoptotic cell death of the cancer cells.

Signaling pathway of telomere shortening-induced replicative senescence and apoptosis in the cancer cells is not fully understood. Telomere shortening may be recognized as DNA damage. This is supported by the fact that in the adenocarcinoma SEG 1 cells, telomerase inhibition was associated with over expression of gene for HR 23B, an early marker of DNA damage [23].

Telomerase suppression and telomere shortening was also associated with up-regulation of genes for p73, p63 of the p53 gene family. Expression of p63 was also confirmed by western blot analysis. Although P53 is frequently mutated in cancer, p73 and p63 are rarely affected. Unlike p53, p73 and p63 exist in different isoforms that may have different targets and opposing effects on replicative senescence and apoptosis. For example, whereas full length form called p73α may induce cell cycle arrest and apoptosis, its truncated isoform AN-p73 actually inhibits cell cycle arrest and apotosis. p73 and p63, like p53, remain localized to the cell nucleus and exert their effects by regulating expression of other genes such as those that mediate cell cycle arrest and apoptosis. Although the precise regulation of p53 family members is not fully understood, it is clear that expressions of p53, p73 and p63 are differently regulated. P73 and p63 share many but not all target genes with p53. Furthermore, the genetic alterations associated with DNA damage are complex and involve genes other than those in the p53 family only that may also influence replicative senescence and apoptosis. For example, E2F1 may induce apoptosis through p53-independent mechanisms. These studies suggest that inhibition of telomerase causes loss of telomeres; loss of telomeres is sensed as DNA damaged by DNA damage sensor such as HR23B; this through other intermediary proteins may lead to up-regulation of p73, p63, E2F1, and mdm2 (see Additional file 1). It has also been shown that in response to DNA damage, E2F1 is stabilized which subsequently upregulates p73. Similarly mdm2 which binds with p53 to inhibit its activity, infact positively regulates p63 and probably also p73.

Up-regulation of p73, p63 and E2F1 may mediate replicative senescence. Cyclin dependent kinase inhibitor, p21 is a key mediator of cell cycle arrest and is a target for p53. Senescence associated with telomere shortening during ageing is accompanied by p53 mediated p21 upregulation. However, p73, p63 and E2F1 have also been shown to cause p53 independent up-regulation of p21. Therefore, it appears that telomerase suppression in SEG- 1 cancer cells leads to induction of p73, p63 and E2F1, which may up-regulate p21 leading to cell cycle arrest and senescence. In addition p16 and GAAD45 were also upregulated in the telomerase siRNA treated cells. Both p16 and GAAD45 are potent inhibitors of cell cycle and contribute to replicative senescence in the cancer cells

In our studies telomerase suppression in SEG1 cells was also associated with up-regulation of genes for FasL, Fas and caspase 8, suggesting that in these cells telomere shortening leads to activation of death receptor mediated apoptosis. P73 can upregulate several genes whose products participate in mitochondrial apoptotic pathway. We found upregulation of caspase recruitment domain containing protein 9 (CARD9) that has been implicated in apoptosis through interaction with Bcl10 and activation of NFKB [24]. We also found that there was upregulation of genes for caspase7 and caspase 3 which are executioner caspases that are implicated in the cleavage of PARP in the final stages of apoptosis. Consistent with these data, a marked induction in the appearance of cleaved (89 kDa) PARP fragment (Figure 5B) was seen in telomerase siRNA treated adenocarcinoma cells. Western blot analysis also indicated a substantial upregulation of TRAIL, which induces apoptosis in cancer cells but not in normal cells.

The gene expression studies provide only an overview of changes in genes involved in the apoptotic pathways. Although for p63, FASL, and p16, the expression changes were confirmed by western blot analyses, further studies of changes in protein expression and activities would be required to fully define the signaling pathways.

In contrast to the effect on the cancer cells, telomerase inhibition did not affect normal somatic cells. This may be due to the fact that normal somatic cells do not express telomerase. Moreover, signaling pathway for these responses in the cancer cells involve p53 homologs p73a, p63a and others that are different from p53-mediated responses in normal cells. Further studies in telomerase expressing normal cells are needed to determine whether effects of the telomerase siRNA are selective for cancer cells.

## Conclusion

Replicative senescence and apoptosis constitute the "failsafe" mechanism against cancer development and development of resistance to replicative senescence and apoptosis is "life line" of cancer cells [1]. Disruption of these pathways is a major goal of chemoprevention, treatment, prevention of recurrences and overcoming resistance to chemotherapy in cancers. By selectively inducing both cell cycle arrest and apoptosis in cancer cells but not in normal somatic cells, Inhibitors of telomerase hold great promise as 'safe' anti-cancer therapeutic agents. SiRNA strategy against telomerase may provide a highly effective and selective method of telomerase inhibition. SiRNAs have been shown to be suitable for *in vivo *delivery and utilization [18]. Further studies, however, are needed to investigate the value of telomerase siRNA in chemoprevention and treatment of Barrett's associated adenocarcinoma.

## Methods

### Cell lines

Esophageal adenocarcinoma cell line (SEG-1), derived from Barrett's's-associated adenocarcinoma of the distal esophagus, was obtained from Dr. David Beer, University of Michigan, Ann Arbor, MI. Normal cell strain from stomach and intestine (CRL7869) was purchased from American Type Culture ollection (Rockville, MD).

### SiRNAs

We designed two siRNA duplexes targeting two different regions of the telomerase gene according to Harborth's criteria [25] for choosing sequences and making siRNA duplexes for target mRNAs. SiRNAs with sense-strand sequences 5'-AAATGCGGCCCCTGTTTCTGG-3' (HTERT A) and 5'-AACATGCGTCGCAAACTCTTT-3' (HTERT B) were submitted in a BLAST search of human EST libraries to ensure that other human genes were not targeted. Pre-annealed siRNA duplexes of above sequences were purchased from Dharmacon Research (Lafayette, CO, USA). For optimization of siRNA transfection, different concentrations of Cy-3 labeled control RNA duplexes (Dharmacon) were delivered into cells using effectene (Qiagen Inc., Valencia, CA), superfact (Qiagen), *Trans*IT-TKO Transfection Reagent (Mirus, Madison, WI), as described by the manufacturer or by eectroporation, as reported by us previously [11].

### SiRNA transfection

The cells were maintained in monolayer culture at 37°C in humidified air with 5% CO2 in Dulbecco's Modified Eagle Medium (DMEM) (Sigma chemical, St. Louis, MO) containing 10% fetal bovine serum, as desribed previously [7,11,26]. For optimization of siRNA transfection, different transfection procedures and siRNA concentrations were used to deliver Cy3-labeled control siRNAs into SEG-1 cells. Transfection using TKO Transfection Reagent (Mirus, Madison, WI) and 50 nM siRNA gave the best transfection efficiency and therefore was used in subsequent experiments to study the effect of telomerase siRNAs on telomere length and survival of BEAC cells. Normal (CRL7869) and adenocarcinoma (SEG-1) cells were transfected with mixture of both telomerase specific siRNAs (Tel-siRNAs). Cells transfected with chemically unmodified non-targeting siCONTROL siRNA (Cont-siRNA) served as a control for non-sequence-specific effects of these molecules.

### Immunocytochemical detection of telomerase

The cells were evaluated for telomerase expression at day 7 following transfection. Cytospins of cells transfected with control or telomerase siRNAs were fixed in methanol/acetone (1:1, v/v) for 10 min at -20°C. Fixed cells were rinsed, rehydrated in PBS, and incubated for 2 h at RT with rabbit polyclonal antibody to telomerase (Novus Biologicals, Inc., Littleton, CO). Antigen-antibody complex was detected by incubation with a fluorescein isothiocyanate (FITC)-labeled secondary antibody.

### Western Blotting

Approximately 50 mg of protein was suspended in Laemmli's sample buffer (0.1 M Tris-HCl buffer pH 6.8, 1% SDS, 0.05% β-mercaptoethanol, 10% glycerol, and 0.001% bromphenol blue), boiled for 2 minutes, and electrophoresed on 4–20% glycerol gradient SDS-acrylamide gel for 4 h at 120 V. Gels were electroblotted onto Trans-Blot nitrocellulose membrane (membrane; Bio-Rad Laboratories, Hercules, CA) at 40 V for 3 h in a Tris-glycine buffer system. Incubation with indicated antibodies was performed for 2 h in PBS-Tween 20 (PBST) containing 1% BSA with constant rocking. Blots were washed with PBST and incubated in either anti-rabbit or anti-mouse horseradish peroxidase (HRPO) conjugates for 2 h in PBST containing 3% nonfat dry milk. After washing, specific proteins were detected using an enhanced chemiluminescence, according to the instructions provided in the manual (Amersham Life Sciences Inc., Arlington Heights, IL).

### Assay of telomerase activity

Telomerase activity was assayed using a fluorescence-based TRAPEZE^R ^XL telomerase detection kit (Intergen, Purchase, NY). TRAPEZE^R ^XL telomerase detection kit provides a refined and fluorometric version of the original TRAP (Telomeric Repeat Amplification Protocol) assay. The kit utilizes "Fluorescence Energy Transfer" primers to generate fluorescently labeled TRAP products and thus allows a highly sensitive and quantitative non-isotopic detection of telomerase activity *in vitro*. Lysates (1000 cell-equivalents) are mixed with TRAPEZE^R ^XL reaction mix containing Amplifuor™ primers, and incubated at 30°C for 30 minutes. Amplified telomerase products are quantitated with a fluorescence plate reader. Telomerase activity (in TPG units) is calculated by comparing the ratio of telomerase products to an internal standard for each lysate, as described by the manufacturer.

### Growth kinetics

SEG-1 cells (5 × 10^5 ^per dish) were transfected with control or telomerase-specific siRNAs, as described above. The transfected cells were harvested by trypsinization, counted and replated weekly at the same live cell number. Prior to re-plating, portions of cells were removed for telomere length, gene expression, and apoptosis/senescence assays.

### Estimation of telomere length

#### Gel-based analysis

For gel-based ananysis of median telomere length, genomic DNA was isolated from telomestatin-treated cells using "Puregene" DNA isolation kits (Gentra Systems, Minneapolis, MN) and telomere length was estimated utilizing the "TeloTAGGG Telomere Length Assay" (Roche Diagnostics Corporation, Roche Applied Science, Indianapolis, IN). In brief, 6 μg of genomic DNA was digested with a sixfold excess of restriction enzymes *Hin*fI and *Rsa*I. Digested DNA, along with a size standard (1-kb DNA ladder), was electrophoresed on 0.8% agarose gel and transferred to Hybond-N+ nylon membrane (Amersham Biosciences Corp, Piscataway, NJ) as described by the manufacturer. Membranes were then hybridized to a digoxigenin (DIG)-labeled telomere-specific probe and telomeric DNA was detected by incubation with alkaline phosphatase coupled to anti-DIG antibody, followed by washing and reaction with a chromogenic substrate of alkaline phosphatase.

#### Telomere-FISH

Cells were treated with KaryoMax (Gibco), hypotonic solution and fixed in glacial acetic acid – ethanol (1:3). For the direct comparison of samples, cell suspensions in the fixative were droped onto a single slide using the 8-well applicator (the plastic top of CultureSlide, Becton Dickinson Labware, Bedford, MA), dropping each sample to the designated well. The air-dried specimens were than processed under virtually identical hybridization conditions, under one coverslip. Q-FISH was done using the Cy3-PNA (C_3_TA_2_)_3 _probe (Applied Biosystems/ BostonProbes, Bedford, MA) as described by Lansdorp [27]. Images were acquired using a 60×/1.40 PlanApo Nikon objective, Nikon Eclipse E6000 microscope equipped with the SD-300-V Optical Head, and Spectral Acquisition v.2.0 software (Applied Spectral Imageing, Vista, CA). Eighty nuclei were analyzed per each sample. The length of a telomere is directly related to its integrated fluorescence intensity value. The quantification of probe signals was done by FISHView v.2.1.1 software (Applied Spectral Imageing) according to manufacturers' recommendations.

### Senescence Assay

Cells were rinsed three times with PBS and fixed in 2% formaldehyde and 0.2% glutaraldehyde solution in in PBS. The cells were washed again as described above and stained overnight in solution containing 1 mg/ml X-gal, 40 mM citric acid/sodium phosphate (pH 6), 5 mM potassium ferrocyanide, 150 mM NaCl, and 2 mM MgCl_2 _[28]. Next day, the stain was removed, cells were rinsed with PBS and staining was viewed under an Olympus^® ^fluorescence microscope.

### Apoptosis Assay

Apoptotic cells were detected using an Annexin V-Biotin Apoptosis Detection Kit (Oncogene Research Products, San Diego, CA). SiRNA-treated myeloma cells (1 × 10^6 ^cells per ml) were mixed with annexin V-biotin and media binding reagent and incubated in the dark for 15 min at room temperature (RT). Cells were then centrifuged and medium was replaced with 1 × Binding Buffer (Oncogene Research Products, San Diego, CA) containing fluorescein isothiocyanate(FITC)-streptavidin (Amersham). Propidium iodide (PI) was added to discriminate early apoptotic cells from late apoptotic or necrotic cells. A portion of cell suspension (50 μl) was placed onto a glass slide, covered with a cover slip and viewed immediately using a fluorescence microscope equipped with FITC (green) and PI (red) filters. Two hundred cells, representing at least five distinct microscopic fields, were analyzed to assess the fraction of FITC and PI-labeled cells for each sample.

### Gene Expression Analysis

Total RNA was isolated utilizing an "RNeasy" kit (Qiagen Inc., USA) and gene expression profile was evaluated using HG-U133 array (Affymetrix, Santa Clara, CA) representing ~33,000 human genes as described previously [7]. GeneChip^® ^arrays were scanned on a GeneArray^® ^Scanner (Affymetrix, Inc., Santa Clara, CA). Array normalization, expression value calculation and clustering analysis were performed using the dChip Analyzer. The Invariant Set Normalization method was used to normalize arrays at probe level to make them comparable, and the model-based method was used for probe selection and to compute expression values [29,30]. These expression levels were assigned standard errors based on replicates, which were subsequently used to compute 90% confidence intervals of fold changes in inter-group comparisons. The lower confidence bounds of "fold change" were conservative estimates of the actual changes. Expression of key genes involved in DNA damage sensing, repair, cell cycle arrest and apoptosis was analyzed.

## Authors' contributions

MAS made the experimental plan, designed siRNAs, carried out telomerase assays, gel-base telomere length analyses, gene expression studies, analyzed the data, and prepared the manuscript. HK carried out cell culture studies, participated in siRNA transfections, performed apoptosis and senescence assays, and helped in the analysis and interpretation of data. RBB assisted in western blot analyses and helped in drafting and critical revision of the manuscript. RCB conducted western blot analyses for telomerase, PARP, and proteins implicated in apoptosis and cell cycle arrest, and also helped in data analysis and preparation of the revised manuscript. AP carried out Telomere-Fish analyses and participated in the data analysis. NCM participated in the design of the study and helped in drafting and critical revision of the manuscript. RKG envisioned the study, and participated in its design and coordination and also helped to draft the manuscript. All authors have read and approved the final manuscript.

## Supplementary Material

Additional File 1 **Gene expression profile following treatment of SEG-1 cells to telomerase specific siRNAs. **SEG-1 cells, transfected with control (Cont) or telomerase specific (Tel) siRNAs were cultured for two weeks and analyzed for gene expression. Total RNA was isolated and hybridized to Human Genome U133 (Affymetrix) representing approximately 33,000 human genes. Expression values of independently conducted experiments showed excellent correlation (R^2 ^0.98), indicating reproducibility of microarray analyses. A subset of DNA damage recognizing, cell cycle checkpoint and apoptosis genes with ≥ 2-fold change in expression is shown. NC = no change Click here for file

## References

[B1] Schmitt CA (2003). Senescence, apoptosis and therapy – cutting the lifelines of cancer. Nat Rev Cancer.

[B2] Blackburn EH (1992). Telomerases. Ann Rev Biochem.

[B3] Shay JW, Wright WE (2004). Senescence and immortalization: role of telomeres and telomerase. Carcinogenesis.

[B4] Tominaga K, Olgun A, Smith JR, Pereira-Smith OM (2002). Genetics of cellular senescence. Mech of Ageing & Dev.

[B5] Kim NW, Piatyszek MA, Prowse KR, Harley CB, West MD, Ho PL, Coviello GM, Wright WE, Weinrich SL, Shay JW (1994). Specific association of human telomerase activity with immortal cells and cancer. Science.

[B6] Herbert BS, Pongracz K, Shay JW, Gryaznov SM, Shea-Herbert B (2002). Oligonucleotide N3' -->P5' phosphoramidates as efficient telomerase inhibitors. Oncogene.

[B7] Shammas MA, Koley H, Beer DG, Li C, Goyal RK, Munshi NC (2004). Growth arrest, apoptosis and telomere shortening of Barrett's associated adenocarcinoma cells by a telomerase inhibitor. Gastroenterology.

[B8] Incles CM, Schultes CM, Kempski H, Koehler H, Kelland LR, Neidles S (2004). A G-quadruplex telomere targeting agent produces p16-associated senescence and chromosomal fusions in human prostate cancer cells. Mol Cancer Ther.

[B9] Stiewe T (2001). p73alpha in apoptosis. Apoptosis.

[B10] Gong JG, Costanzo A, Yang HQ, Melino G, Kaelin WG, Levrero M, wang JY (1999). The tyrosine kinase c-abl regulates p73 in apoptotic response to cisplatin-induced DNA damage. Nature.

[B11] Shammas MA, Simmons CG, Corey DR, Shmookler Reis RJ (1999). Telomerase inhibition by peptide nucleic acids reverses 'immortality' of transformed human cells. Oncogene.

[B12] Barma DK, Elayadi A, Falck JR, Corey DR (2003). Inhibition of telomerase by BIBR1532 and related analogues. Bioorg Med Chem Lett.

[B13] Vonderheide RH, Domchek SM, Schultze JL, George DJ, Hoar KM, Chen DY, Stephomi KF, Masutomi K, Loda M, Xia Z, Anderson KS, Hahn WC, Nadler LM (2004). Vaccination of cancer patients against telomerase induces functional antitumour CD^+ ^T lymphocytes. Clin Cancer Res.

[B14] Spechler SJ, Goyal RK (1986). Barrett's esophagus. N Engl J Med.

[B15] Devesa SS, Blot WJ, Fraumeni JF (1998). Changing patterns in the incidence of esophageal and gastric carcinoma in the United States. Cancer.

[B16] Lord RV, Salonga D, Danenberg KD, Peters JH, DeMeester TR, Park JM, Johansson J, Skinner KA, Chandrasoma P, DeMeester SR, Bremner CG, Tsai PI, Danenberg PV (2000). Telomerase reverse transcriptase expression is increased early in the Barrett's metaplasia, dysplasia, adenocarcinoma sequence. J Gastrointest Surg.

[B17] Shammas MA, Shmookler Reis RJ, Akiyama M, Koley H, Chauhan D, Hideshima T, Goyal RK, Hurley LH, Anderson KC, Munshi NC (2003). Telomerase Inhibition and Cell Growth Arrest by G-Quadruplex Interactive Agent in Multiple Myeloma. Mol Cancer Ther.

[B18] Song E, Lee SK, Wang J, Ince N, Ouyang N, Min J, Chen J, Shankar P, Lieberman J (2003). RNA interference targeting Fas protects mice from fulminant hepatitis. Nat Med.

[B19] Harley CB, Futcher AB, Greider CW (1990). Telomeres shorten during aging of human fibroblasts. Nature.

[B20] Shammas MA, Shmookler Reis RJ (1999). Recombination and its roles in DNA repair, cellular immortalization and cancer. Age.

[B21] Shay JW, Roninson IB (2004). Hallmarks of senescence in carcinogenesis and cancer therapy. Oncogene.

[B22] Murphy JF, McGregor JL, Leung LL (1998). Senescent human neutrophil binding to thrombospondin (TSP): evidence for a TSP-independent pathway of phagocytosis by macrophages. Br J Haematol.

[B23] Volker M, Mone MJ, Karmakar P, van Hoffen A, Schul W, Vermeulen W, Hoeijmakers JHJ, van Driel R, van Zeeland AA, Mullenders LHF (2001). Sequential assembly of the nucleotide excision repair factors in vivo. Molec Cell.

[B24] Bertin J, Guo Y, Wang L, Srinivasula SM, Jacobson MD, Poyet JL, Merriam S, Du MQ, Dyer MJ, Robison KE, DiStefane PS, Alnemri ES (2000). Card9 is a novel caspase recruitment domain-containing protein that interacts with BCL10/CLAP and activates NF-kappa B. J Biol Chem.

[B25] Harborth J, Elbashir SM, Bechert K, Tuschl T, Weber K (2001). Identification of essential genes in cultured mammalian cells using small interfering RNAs. J Cell Sci.

[B26] Shammas MA, Raney KD, Subramanian S, Shmookler Reis RJ (2004). Telomere length, cell growth potential, and DNA integrity of human immortal cells are all compromised by peptide nucleic acids targeted to the telomere or telomerase. Experimental Cell Research.

[B27] Lansdorp PM, Verwoerd NP, van de Rijke FM, Dragowska V, Little MT, Dirks RW, Raap AK, Tanke HJ (1996). Heterogeneity in telomere length of human chromosomes. Hum Mol Genet.

[B28] Dimri G, Lee P, Basile X, Acosta G, Scott M, Roskelley G (1995). A biomarker that identifies senescent human cells in culture and in aging skin in vivo. Proc Natl Acad Sci USA.

[B29] Li C, Hung Wong W (2001). Model-based analysis of oligonucleotide arrays: model validation, design issues and standard error application. Genome Biol.

[B30] Li C, Wong WH (2001). Model-based analysis of oligonucleotide arrays: expression index computation and outlier detection. Proc Natl Acad Sci USA.

