# Supplementary material for: Telomerase inhibition by siRNA causes senescence and apoptosis in Barrett's adenocarcinoma cells: mechanism and therapeutic potential
Source: Mol Cancer. 2005 Jul 15;4:24. doi: 10.1186/1476-4598-4-24 (PMC1187920; doi:10.1186/1476-4598-4-24)
Supplement: Additional File 1 — Gene expression profile following treatment of SEG-1 cells to telomerase specific siRNAs. SEG-1 cells, transfected with control (Cont) or telomerase specific (Tel) siRNAs were cultured for two weeks and analyzed for gene expression. Total RNA was isolated and hybridized to Human Genome U133 (Affymetrix) representing approximately 33,000 human genes. Expression values of independently conducted experiments showed excellent correlation (R2 0.98), indicating reproducibility of microarray analyses. A subset of DNA damage recognizing, cell cycle checkpoint and apoptosis genes with ≥ 2-fold change in expression is shown. NC = no change [file 1476-4598-4-24-S1.doc]

Continued on next page - - -
